# Automatic Facial Paralysis Assessment via Computational Image Analysis

**DOI:** 10.1155/2020/2398542

**Published:** 2020-02-08

**Authors:** Chaoqun Jiang, Jianhuang Wu, Weizheng Zhong, Mingqiang Wei, Jing Tong, Haibo Yu, Ling Wang

**Affiliations:** ^1^Shenzhen Institutes of Advanced Technology, Chinese Academy of Sciences, Beijing, China; ^2^Hohai University, Jiangsu, China; ^3^Shenzhen Traditional Chinese Medicine Hospital, Guangdong, China; ^4^Nanjing University of Aeronautics and Astronautics, Nanjing, China

## Abstract

Facial paralysis (FP) is a loss of facial movement due to nerve damage. Most existing diagnosis systems of FP are subjective, e.g., the House–Brackmann (HB) grading system, which highly depends on the skilled clinicians and lacks an automatic quantitative assessment. In this paper, we propose an efficient yet objective facial paralysis assessment approach via automatic computational image analysis. First, the facial blood flow of FP patients is measured by the technique of laser speckle contrast imaging to generate both RGB color images and blood flow images. Second, with an improved segmentation approach, the patient's face is divided into concerned regions to extract facial blood flow distribution characteristics. Finally, three HB score classifiers are employed to quantify the severity of FP patients. The proposed method has been validated on 80 FP patients, and quantitative results demonstrate that our method, achieving an accuracy of 97.14%, outperforms the state-of-the-art systems. Experimental evaluations also show that the proposed approach could yield objective and quantitative FP diagnosis results, which agree with those obtained by an experienced clinician.

## 1. Introduction

Facial paralysis (FP, also known as peripheral facial nerve paralysis) occurs on one or both sides of the face when cranial nerve number 7 is injured. Such an injured nerve, being originally responsible for several functions in the face, is not able to control muscles for normal facial motions [1, 2]. Therefore, facial asymmetry would commonly appear in FP patients that cause significant inconveniences to their daily life (e.g., work and social communication). Timely and effective treatment of FP can alleviate the facial disfigurement, where an objective diagnosis of FP plays an important role in the whole procedure of treatment.

### 1.1. Related Work

#### 1.1.1. Subjective Assessment

House–Brackmann (HB) facial nerve grading system [3] is a representative scaling method for subjectively assessing FP. It has been adopted by the American Academy of Otolaryngology-Head and Neck Surgery since 1985. In recent years, more scaling methods such as Hato [4], Sunnybrook [5], and FDI [6] have appeared and been used in the clinic. These kinds of subjective assessment systems are easy to use. However, they rely largely on the clinician's subjective observations from the patient's specific facial movements and grading tables. As a consequence, this operation not only is a time-consuming and labor-intensive procedure but also is an individual expertise dependent.

#### 1.1.2. Objective Assessment

Objective assessment of FP has appeared recently. The first type of these assessment methods is based on the electromyography signals, such as electromyography (EMG) [7], surface electromyography (sEMG) [8], and Electroneuronography (ENoG) [9], which can detect the state of the entire facial motor system and determine the degree of facial nerve damage. EMG [7] needs to puncture small needles into special facial muscles, and the patient will be asked to exercise these muscles to record nerve signals. Thus, EMG is invasive which leads to a complicated operation. The improved sEMG [8] is more convenient to operate by using patch electrodes. However, in actual operations, the electrodes are hardly placed uniformly, and much additional interference would be introduced. ENoG [9] could judge the degree of peripheral facial nerve damage by applying electrical stimulation to the facial nerve trunk from the stem pore and recording electrophysiological parameters from the facial muscles of the distal part.

Facial paralysis grading can benefit from the recent development of computer vision. Wachtman et al. [10] recorded facial motion tasks (brow raise, eye closure, and smile) of patients by image tracking. He et al. [11] evaluated 197 cases of facial paralysis images using an automatic evaluation system based on the local binary pattern related to biomedical image recognition technology, with a classification accuracy of 94%. Guo et al. [12] collected the facial expression images of patients with a known HB score. They detected the face landmark points of the images and then obtained structural features. A support vector machine (SVM) classifier is generated using these structural features, which can automatically assess the HB score of FP patients. Recently, Guo et al. [13] utilized a deep convolutional neural network for feature extraction and degree prediction in the unilateral peripheral facial paralysis assessment.

However, the accuracy of computer vision-based objective facial paralysis assessment is not good enough because the difference between images of facial paralysis patients with different HB levels is too small to distinguish [12]. A lot of studies have illustrated that, once certain lesions occur in the human bodies, the tissue metabolism and blood circulation on the lesion areas will change, resulting in the fluctuations of local temperature on the body surface and abnormal infrared heat radiation. Liu et al. [14, 15] evaluated facial paralysis using an infrared thermal image, which localizes facial features based on both the specificity of facial temperature distribution and the image edge detection technique and divides the image into eight regions. Then, the features and asymmetry degree of facial temperature distribution are extracted automatically, and finally, the radial basis function neural network is employed as the automatic classifier to assess the HB score of FP patients. However, there are many factors that can distort the infrared thermal image feature, e.g., fever and some outside influence, which let the accuracy of the method be reduced.

According to Cui et al. [16], the facial skin perfusion will be affected after the onset of Bell's palsy. A possible connection between facial skin microcirculation and Bell's palsy may be the edema surrounding facial nerve, which is often found in facial paralysis patients in decompression operation and magnetic resonance imaging. The edema will result in elevation of pressure and further damage. Since the direction of facial nerve blood flow is primarily proximal to distal [17, 18], the facial skin microcirculation near the facial nerve would also be affected.

Our proposed approach follows the similar hypothesis proposed by Cui et al. [16] that the facial perfusion of Bell's palsy patients can reflect the stage of Bell's palsy. Laser speckle contrast imaging (LSCI) is applied in this study, which is an effective real-time, full-area blood flow imaging technique. It has attracted extensive attention in the fields of biomedical imaging and clinical diagnosis. Dunn et al. [19, 20] used LSCI to monitor the changes in cerebral blood flow. Kashima and Hayashi [21] applied LSCI to observe the effects of different tastes on facial blood flow. Tian et al. [22] employed LSCI to compare the effects of the metal needle and the laser needle on facial blood perfusion. Finsterer [23] utilized LSCI to study the treatment effect of acupuncture on FP patients from the blood flow of ocular skin. Cui et al. [16, 24] observed that the blood perfusion of the affected facial side of most FP patients was lower than the healthy side and was positively related to the severity of facial paralysis.

In this paper, we propose an efficient yet objective facial paralysis assessment approach via automatic computational image analysis. After capturing both the blood flow and RGB images of the patient's faces by LSCI scanners, the patient's face is first divided into different regions to extract facial blood flow distribution characteristics. Then, machine learning-based HB score classifiers are trained using a large number of data of facial blood flow distribution characteristics. Finally, the HB score classifiers of K-nearest Neighbor (K-NN), SVM, and Neural Network (NN) could produce good prediction accuracy in our experiments.

### 1.2. Overview of the Proposed Approach

Our approach of computational image analysis serves the HB grading system. We can make such a subjective system be an automatic and objective assessment tool for a more accurate FP diagnosis. The HB grading system was first introduced by House and Brackmann in 1985 [3] and then was commonly used in clinical environments. Facial paralysis patients can be scored from I to VI to describe the facial nerve function from normal to no movement. As shown in Table 1, the larger the score is, the more serious the damage of the facial nerve function is. In our study, the HB score is also used for the assessment of facial paralysis.

Our facial paralysis assessment approach is based on the hypothesis that the stage of Bell's palsy could be measured by the facial skin perfusion. There are many causes of Bell's palsy, including virus HSV-1, ischemia of the facial nerve, and some uncertain factors [2]. Besides, facial nerve swelling is a presence in the reported decompression operations and contrast-enhanced magnetic resonance imaging in Bell's palsy patients [23, 25]. The edema surrounding facial nerve will result in elevation of pressure, ischemia of the facial nerve, and further damage. Since the facial nerve blood flow is primarily proximal to distal, the microcirculation of distal tissue, such as skin, could be affected if the microcirculation of tissue near the facial nerve changes. According to Cui et al. [16], although the reason why facial skin perfusion is affected by Bell's palsy needs further investigation, and the phenomenon that facial skin perfusion changes after the onset of Bell's palsy was evidenced by their research. They mainly recommend doing further research on finding the connection between the facial skin perfusion changes and Bell's palsy for objective assessment of Bell's palsy.

**Figure 1:**
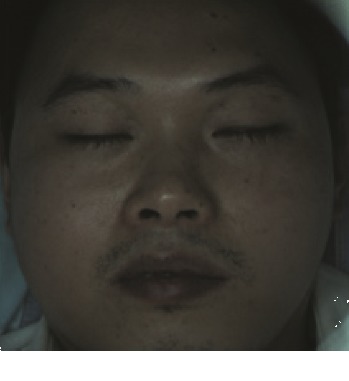
The proposed framework of the automatic facial paralysis assessment system based on quantitative computational image analysis.

Our designed assessment system focuses on the patients with unilateral Bell's palsy in the acute stage [23]; more specifically, the FP occurs within 7 days. In summary, the overview of our method is presented in Figure 1. For automatic assessment of FP, measurement of patients' facial skin perfusion is firstly needed, for which the LSCI technique is quite fit. Then, the facial skin perfusion is analyzed by an improved facial blood image segmentation method. Since a pair of pixel-to-pixel color image and blood flow image is measured simultaneously by the LSCI device, it is much easier to segment facial regions using the color image. 3D face reconstruction from a single image is a fundamental problem in computer vision and graphics, which can be applied to segment the concerned face regions efficiently and precisely. In this approach, the facial landmark points are estimated first. Then, a reconstruction 3D face model can be fitted using a 3D morphable face model and those facial landmark points. Along with the reconstruction 3D face model, a transformation matrix can be calculated, which could map the vertex in the 3D model to the 2D pixel in the color image. Because the vertex order in the 3D face model is well known, some concerned facial regions are marked in advance, and those regions can map to color image using the transformation matrix.

Finally, the facial blood flow distribution characteristics are obtained from the regional mean blood volume calculation operation. The HB score classifiers are trained for assessment by using a large amount of facial blood flow distribution characteristic data. Three well-known classifiers, namely, K-NN, SVM, and NN, are employed for assessment.

## 2. Methods

### 2.1. Facial Blood Flow Measured by LSCI

**Figure 2:**
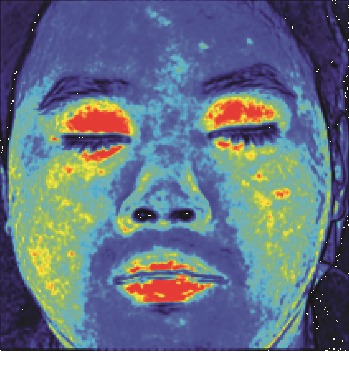
(a) Color image. (b) Color blood flow image. (c) Gray blood flow image.

In this study, the LSCI technology is used to measure facial skin microcirculation perfusion distribution of Bell's palsy patients. LSCI technology, which is harmless to participants, is a noninvasive optical-based measuring technique for microcirculation perfusion. The tissue being studied is illuminated by a near symmetrical laser beam, and the light scattered by the particles in the illuminated tissue forms an interference image and is captured by the camera [19]. These particles fluctuate as the blood flows, and when captured by the camera, they integrate into a dynamic speckle image of blood flow. Higher blood flow causes more particle motion during the camera's integration time, leading to more blurred interference images. By measuring the degree of blurring of the interference image, LSCI can generate a real-time full-field perfusion distribution image. A blood flow image is commonly rendered using window width and window level treatment algorithm. A sample of the LSCI image in our method is shown in Figure 2.

### 2.2. Facial Blood Image Segmentation

We design a facial blood image segmentation method, as the preprocessing section of the HB score classifier, to automatically segment the facial blood flow image to obtain blood flow information. The LSCI device outputs the blood flow image and color image simultaneously, and the two images can be matched pixel-by-pixel. In the facial blood image segmentation method, we reconstruct a 3D face model using the facial color image and the Surrey face model [26] with a 3D morphable face model reconstruction algorithm. Then, facial blood flow distribution characteristics are obtained by calculating the average blood flow volume of concerned facial regions.

Surrey face model is a 3D morphable face model [26], which consists of a principal component analysis (PCA) model of the face shape and color information and allows to reconstruct a 3D face model from a single 2D image. The PCA model $M≔\left(\bar{v},\sigma ,V\right)$ consists of the components $\bar{v}\in {\mathbb{R}}^{3\times N}$, where *N* is the number of model vertices, a set of principal components *V*=[*v*_1_, *v*_2_,…, *v*_*n*−1_] ∈ *ℝ*^3×*N*×(*n* − 1)^, and the standard deviations *σ* ∈ *ℝ*^*n*−1^. When given a principal coefficient *α* ∈ *ℝ*^*M*^ and *M* ≤ *n* − 1, a new face model can be created by resolving $S=\bar{v}+\sum_{i}^{M}\alpha_{i}\sigma_{i}v_{i}$.

The first step of facial blood image segmentation is face landmark fitting. The main task of this step is to obtain the 2D coordinates of the face landmarks from the input color image. Set the input color image as *I*_color_ and the output 2D points as *p*_*i*_ ∈ *ℝ*^2^,  *i*=0,1,…, 67, and an ensemble of regression trees are used [27] to regress the location of facial landmarks from the color image *I*_color_.

**Figure 3:**
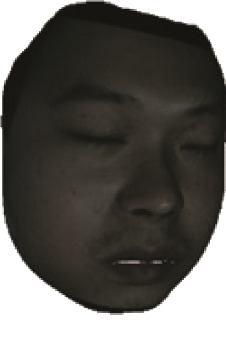
(a) 68 face landmarks. (b) Face landmarks in red color.

The second step is camera estimation using the set of 2D face landmark coordinates *p*_*i*_ ∈ *ℝ*^2^,  *i*=0,1,…, 67, as shown in Figure 3 and their known correspondences in the Surrey face model to estimate the position of the camera model. An affine camera model's matrix can be estimated using the gold standard algorithm [28]. The 2D face landmark points *p*_*i*_ and the corresponding 3D model points are, respectively, represented in homogeneous coordinates *x*_*i*_ ∈ *ℝ*^3^ and *X*_*i*_ ∈ *ℝ*^4^. With the gold standard algorithm, the estimated camera matrix *C* ∈ *ℝ*^3×4^ is available.

The third step is 3D model fitting using the estimated camera matrix *C* and Surrey face model's PCA shape model. We modify the PCA shape model's coefficients to fit the 3D model with the 2D face landmarks, resorting to the shape-to-landmark fitting technique [29]. When the following cost function (1) reaches the minimum, the coefficients *α* ∈ *ℝ*^*M* ^ are achieved:

(1) $$\begin{array}{c} \begin{array}{c} E=\overset{N}{\underset{i=1}{\sum }}\frac{{\left(y_{m2\text{D},i}-x_{i}\right)}^{2}}{2\sigma_{2\text{D}}^{2}}+\left\|\alpha_{2}^{2}\right\| \end{array}, \end{array}$$

where *N* is the number of face landmarks, in our case *N*=68, *x*_*i*_ are 2D face landmarks in homogeneous coordinates, *y*_*m*2D,*i*_ are the projections of the corresponding 3D model points using the estimated camera matrix *C*, and *σ*_2D_^2^ is an optional variance for the 2D face landmark points. We set the index of the corresponding 3D model points as *h*, and $y_{m2\text{D},i}=C\cdot {\left(\hat{V_{h}}\alpha +{\bar{v}}_{h}\right)}_{\text{homogeneous coordinate}}$, where *V*=[*v*_1*h*_, *v*_2*h*_,…, *v*_*n*−1*h*_]. Therefore, the estimated PCA coefficients *α* can be obtained by solving a linear system of equations, which can generate the final face model by $S=\bar{v}+\sum_{i}^{M}\alpha_{i}\sigma_{i}v_{i}$.

**Figure 4:**
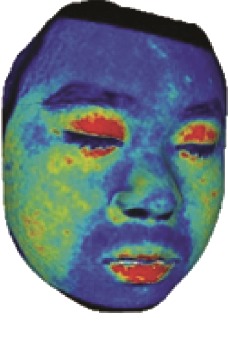
3D face model (a) with color texture, (b) with facial blood texture, and (c) with premarked regions.

**Figure 5:**
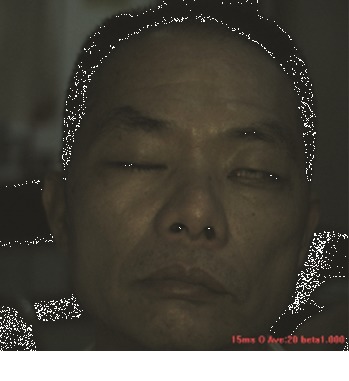
The premarked regions used in the quantitative assessment. L means left side, R means right side, and other abbreviations are listed in Table 2.

The fourth step is the regional segmentation. For each vertex *v*_*i*_ in face model *S*, the corresponding 2D point *p* in the input image *I*_color_ is *p*=*C* · *v*. Because the facial blood image is matched with color image pixel-to-pixel, *p* is also the correct point in blood flow image *I*_blood_. The reconstructed 3D face model is shown in Figure 4. Since the vertex order of the PCA model is already known, we premark some regions of interest on the 3D face model. These premarked regions include eye circumference, eyebrow, cheek, nose wing, mouth upper, mouth below, and mouth corner. The premarked regions are illustrated in Table 2 and Figure 5. The facial blood flow distribution characteristics are achieved by calculating each region's average blood flow volume from the regions matched in blood flow image *I*_blood_.

### 2.3. HB Score Classifier

Through the facial blood image segmentation method, we get the facial blood flow distribution characteristics. The facial blood flow distribution characteristics are, respectively, fed into three commonly used classifiers K-NN, SVM, and NN to provide a quantitative evaluation of facial paralysis. The patients are divided into two groups: left side facial paralysis patients and right side facial paralysis patients. We call the average blood volume of regions in the affected side of the face as *A*_region_ and in healthy side as *H*_region_. As a consequence, the input vector of the HB score classifier is [*A*_B_,  *A*_E_, *A*_N_, *A*_C_, *A*_MU_, *A*_MC_, *A*_MB_, *H*_B_,…, *H*_MB_], and the output result is a six-column vector in which each value means the likelihood of a corresponding HB score. Therefore, the HB score of the facial paralysis patient is estimated using our HB score classifier.

## 3. Experiments

### 3.1. Data Collection

The LSCI device used in our study is developed by Wuhan SIM Opto-Technology Co., Ltd., which can monitor a large area of tissue perfusion by real-time dynamic imaging. The device can collect facial blood flow images and color images of participants simultaneously up to 60 fps with the resolution of 512 × 512. Each pixel in the output facial blood flow image is a positive real number which means the blood flow volume of that pixel.

This study was approved by the Research Ethics Committee for Shenzhen Traditional Chinese Medicine Hospital. All participants were clearly aware of the experimental purposes and procedures. A large amount of LSCI data have been collected from real Bell's palsy patients and some healthy volunteers. Screened by professional clinicians, the selected patients met the diagnostic criteria of Bell's facial paralysis and then were scored by the HB grading system. The valid data used in this paper is a total of 80 people, including 8 healthy people, 17 with score II, 16 with score III, 13 with score IV, 16 with score V, and 10 with score VI. All valid LSCI data were within the acute phase of facial paralysis, which occurs within 7 days.

**Figure 6:**
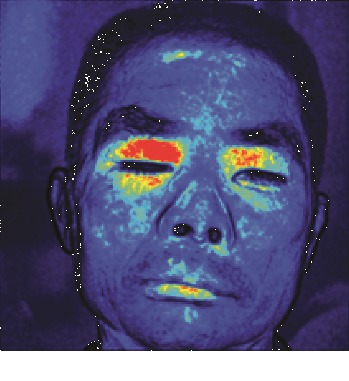
(a) The normal color facial blood flow image. (b) The wrong color blood flow image. The two images were saved during one continuous scanning, but image (b) is slightly blurred due to the movement of the participant's head, and the facial blood flow data are abnormally high.

When collecting the LSCI data, the participants were required to lie on the bed in a calm state, with eyes closed, and facial expressions relaxed naturally. The collecting room was illuminated with natural light and fluorescent lights without direct sunlight. A soft pillow was placed under the participants' head and neck to prevent the participants' head moving. The LSCI device was fixed about 20 cm above the participants' faces to continuously measure facial blood flow images. If the participant's head moved during scanning, the blood flow of the entire face in the blood flow image would increase, resulting in a large data error. The normal LSCI blood flow image and the LSCI image at the time of head shaking are illustrated in Figure 6. All wrong images were deleted in this experiment. Finally, a total of 100 LSCI blood flow images of each participant were collected, resulting in a total of 8,000 LSCI images.

**Figure 7:**
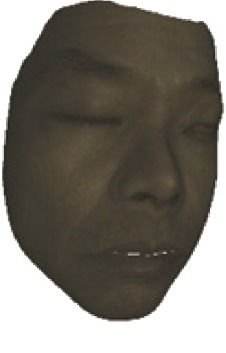
The ratio of the affected side to the healthy side of orbital blood flow in patients with different HB scores. From left to right, the HB score is from I to VI. For the healthy samples with HB score (I), we use the right side as the affected side.

The collected LSCI data of the facial paralysis patients proved the asymmetry of facial blood flow distribution, and the stage of asymmetry was positively related to the grade of facial paralysis. The blood flow value of the healthy side of the ocular skin is marked as *P*_*h*_, and *P*_*a*_ means the blood flow value on the affected side. Let *P*_*r*_=*P*_*h*_/*P*_*a*_; the closer the *P*_*r*_ is to 1, the more symmetric the blood flow perfusion on both sides of the face is. It can be seen from Figure 7 that the degree of difference in *P*_*r*_ of the patient's ocular skin increases with the HB score. There are similar differences in patients' other face regions with the changes in the HB score. Therefore, the degree of facial paralysis of patients can be evaluated by automatically analyzing the blood flow distribution in different regions of the face.

### 3.2. Segmentation Performance Evaluation

In facial blood image segmentation, some concerned regions are extracted, including eyebrow, eye circumference, cheek, nose wing, mouth upper, mouth below, and mouth corner. In order to evaluate the segmentation performance with respect to the accuracy, the Dice's coefficient (DSC) is employed, which is a statistic used to gauge the similarity of two samples and is a commonly used metric in image segmentation. The DSC is defined as follows:

(2) $$\begin{array}{c} \begin{array}{c} \text{DSC}=\frac{2\left|X\cap Y\right|}{\left|X\right|+\left|Y\right|} \end{array}, \end{array}$$

where *X* means the estimated region, while *Y* represents the true answer labeled manually. DSC is the accuracy of the estimated region; the higher it is, the better the segmentation is; DSC=2∑_*i*_^regions^(|*X*_*i*_∩*Y*_*i*_|)/∑_*i*_^regions^(|*X*_*i*_|+|*Y*_*i*_|) is used to describe the accuracy of multiple face regions.

In our study, a skilled clinician had manually labeled 100 facial blood flow images with correct facial segmentation as the ground truth. Comparing the estimated result with the ground truth, the accuracy of our facial blood flow image segmentation method is shown in Table 3.

### 3.3. Automatic HB Score Classifier

After facial blood image segmentation, the average blood flow volume of regions is sorted as [*A*_B_,  *A*_E_, *A*_N_, *A*_C_, *A*_MU_, *A*_MC_, *A*_MB_, *H*_B_,…, *H*_MB_]. The average blood volume of regions in the affected side of the face is *A*_region_, and in the healthy side is *H*_region_. However, since the uncertainties are caused by the complexities of the LSCI device, it can only give microcirculation blood perfusion values in relative units [30]. Therefore, in the actual experiment, the blood flow value difference of different patients is neglected, and the patient's facial blood flow is scaled to obtain a relative value. Find the smallest blood flow value face region's blood flow volume Volume_min_=Min(*A*_B_,  *A*_E_, *A*_N_, *A*_C_, *A*_MU_, *A*_MC_, *A*_MB_, *H*_B_,…, *H*_MB_), and divide the blood flow value of all regions by Volume_min_.Then, get the relative blood flow value [*A*_B_,  *A*_E_, *A*_N_, *A*_C_, *A*_MU_, *A*_MC_, *A*_MB_, *H*_B_,…, *H*_MB_]/Volume_min_ as the actual input vector of our HB score classifier. The corresponding HB scores of patients are gold standards of our LSCI dataset.

In the implementation, the NN model consists of an input layer, two hidden layers, and one output layer. The kernel function of the SVM model is a polynomial function with a degree of two. Euclidean distance is the criterion for evaluating the distance between data in the K-NN model, and the value of *k* is 11. *K*-fold cross-validation method is applied to evaluate the correctness of the three HB score classifiers NN, SVM, and K-NN. The *k*-fold cross-validation method divides the dataset into *k* subsamples, and a single subsample is retained as the data of the verification model, and the other *k* − 1 samples are used for training. Cross-validation is repeated by *k* times, each subsample is verified once, and the average *k*-time results are used to finally obtain a single estimate. The experimental dataset is disordered in units of patients; experimental results of *k*=5,  10 are listed in Table 4.

In the experiment, we found that the NN model achieved an optimal HB classification accuracy of 97.14%, the accuracy of the SVM classifier was acceptable, and the results by the K-NN model were the worst among the three classifiers. In the literature [15], the accuracy of the infrared image analysis method using the radial basis function neural network (RBFNN) classifier is 94.10%. In contrast, our NN model achieves an accuracy of 97.14%, with 3.04% higher than that of the RBFNN classifier.

Finally, the performance of our automatic assessment system is illustrated in Table 5. The color images and blood flow images collected by LSCI and the intermediate results (face reconstruction) in facial blood image segmentation are presented. At the same time, the HB results achieved automatically by the proposed assessment system, and the HB results judged by the experienced clinician are also given, respectively. It can be seen that the automatic assessments highly align with the clinician assessments.

## 4. Discussion

There are many speculations about the cause of facial paralysis. Modern medicine believes that viral infection, ischemia of the facial nerve, and other uncertain factors can lead to FP [1, 2]. As stated in [16], the facial skin perfusion will change after the onset of Bell's palsy. The probable reason is the facial nerve edema, which is often found in FP patients and can result in elevation of pressure, ischemia, and further damage. Since the facial nerve blood flow direction is from the proximal end to the distal end [18], the facial nerve ischemia is reflected in the blood flow microcirculation on the surface of the facial skin [17].

Our novel proposed method is both accurate and efficient because our method directly assesses the FP stage by automatically analyzing the facial skin perfusion from the biomedical facial blood flow image produced by the LSCI device. LSCI technology provides a new blood flow imaging method for clinical diagnosis and basic research of life sciences with its advantages of the large detection area, noncontact type, mark-free, high resolution, and rapid imaging [19, 20]. In our proposed system, the LSCI scanner can measure the blood flow images of the entire facial area of the patient. The patient does not need to be needled like the EMG method, and the monitored blood flow images can be generated real-time. It is, therefore, a convenient tool for both patients and clinicians.

To the best of our knowledge, this is the first study to apply the LSCI images to develop an automated facial paralysis assessment system. In the automated assessment process, LSCI images are used to measure facial microcirculation blood flow of patients with facial paralysis. Since the amount of collected LSCI data in this experiment is relatively small, it is not enough to train a large deep learning network like a deep residual learning network [31]. As a consequence, a facial blood image segmentation method is proposed to be used as a preprocessing step for the HB score classifier. 3D face reconstruction from a single-image method is applied to segment the concerned face regions efficiently and precisely. As shown in Table 3, the segmentation accuracy is excellent, especially the main concerned facial regions, such as eyebrow, eye circumference, and cheek, with accuracy higher than 90%. After this preprocessing step, the input data of the classifier are far simplified. Three HB score classifiers NN, SVM, and K-NN verify the classification performance of the automated evaluation method and achieve excellent classification accuracy up to 97.14%.

## 5. Conclusion

In this paper, we have proposed a novel computational image analysis approach to automatically and quantitatively assess the facial paralysis. First, the facial blood flow of patients with facial paralysis is measured by an LSCI device for generating RGB color images and blood flow images. Second, a facial blood image segmentation method is used as the preprocessing section of the HB classifier. Finally, three machine learning methods including NN, SVM, and K-NN are employed to classify the quantitative degrees of the patient. A large dataset of 8,000 captured images from 80 volunteers is collected, confirming that our novel approach is highly accurate and robust with accuracy up to 97.14%. Therefore, the novel computational image analysis approach can be used as an effective tool for the automatic assessment of facial paralysis.

## Figures and Tables

**Table 1:** The House–Brackmann (HB) grading system.

Score	Description
I	Normal
II	Slight dysfunction
III	Moderate dysfunction
IV	Moderate-to-severe dysfunction
V	Severe dysfunction
VI	Total paralysis

**Table 2:** Description of the premarked regions.

Index	Region	Abbreviation	Scope
1	Eyebrow	B	The small region above the eyebrow
2	Eye circumference	E	The area within the eye socket
3	Nose wing	N	Area on both sides of the nose
4	Cheek	C	Part of the cheek
5	Mouth upper	MU	The small region above the mouth
6	Mouth corner	MC	The small region near the mouth corner
7	Mouth below	MB	The small region below the mouth

**Table 3:** The accuracy performance of our segmentation method measured by DSC.

Region	Accuracy (left) (%)	Accuracy (right) (%)	Accuracy (both) (%)
Eyebrow	91.47	91.42	91.43
Eye circumference	95.87	95.52	95.69
Nose wing	87.85	87.44	87.63
Cheek	97.63	97.03	97.34
Mouth upper	87.53	87.18	87.22
Mouth corner	88.21	87.98	88.15
Mouth below	87.43	87.97	87.75
Total	93.06	94.46	93.98

**Table 4:** The accuracy of three classifiers in terms of cross-validation.

Cross-validation	Neural network (%)	SVM (%)	K-NN (%)
5	96.77	86.77	67.74
10	97.51	87.34	71.35
Mean value	97.14	87.06	69.55

**Table 5:** Performance of our automatic assessment system.

Color image	Facial blood image	Ill side	Color model	Blood model	Estimated HB score/real HB score
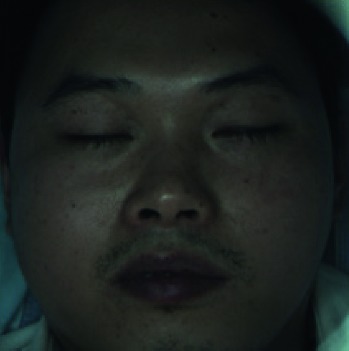	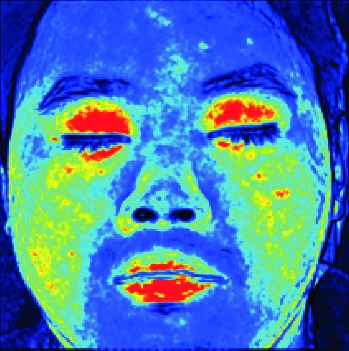	Left	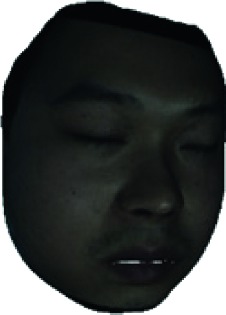	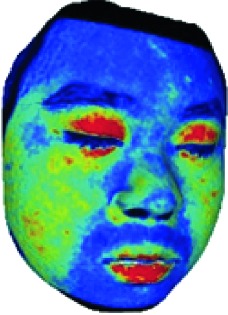	II/II

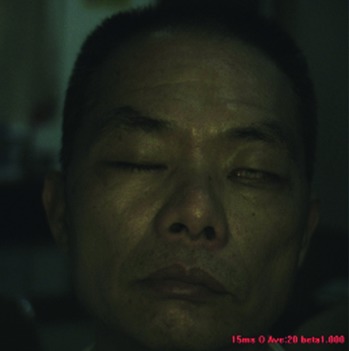	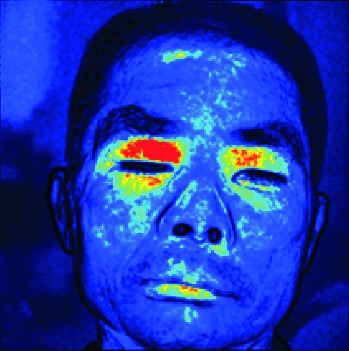	Left	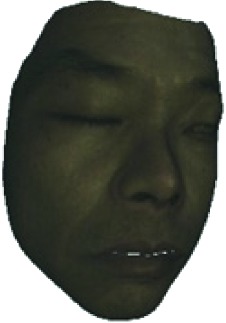	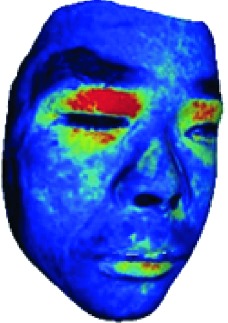	VI/VI

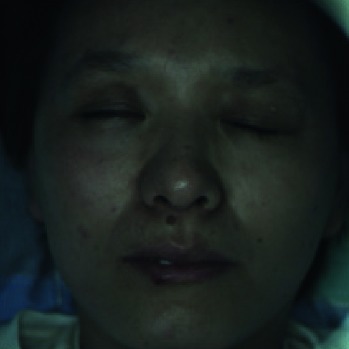	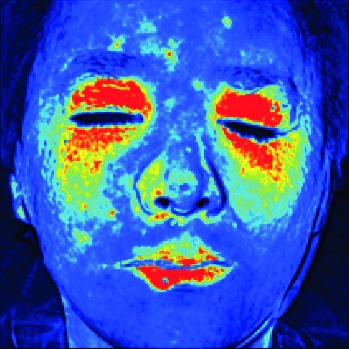 0	Right	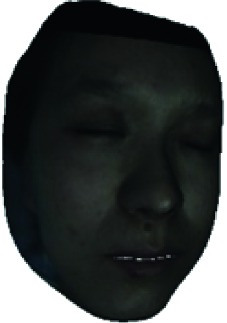	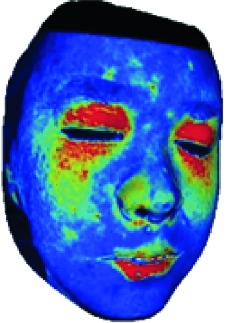	V/V

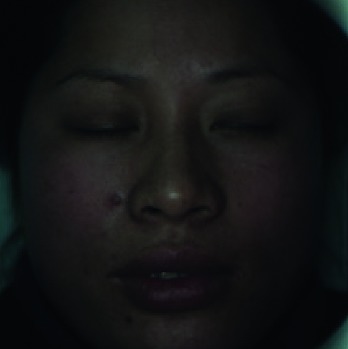	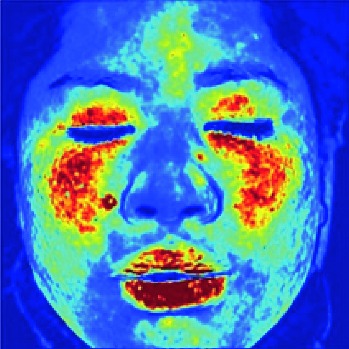	Left	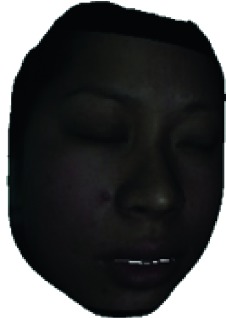	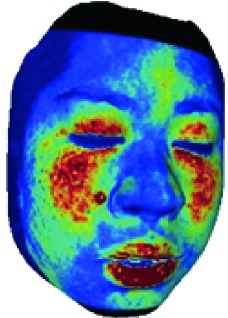	III/III

## Data Availability

The data used to support the findings of this study are not included within the article.
